# Identifying secreted biomarkers of dopaminergic ventral midbrain progenitor cells

**DOI:** 10.1186/s13287-023-03580-5

**Published:** 2023-12-10

**Authors:** Pedro Rifes, Marc Isaksson, Charlotte Rusimbi, Adrián Ramón Santonja, Jenny Nelander, Thomas Laurell, Agnete Kirkeby

**Affiliations:** 1 Novo Nordisk Foundation Center for Stem Cell Medicine – reNEW, University of Copenhagen, Blegdamsvej 3B, 2200 Copenhagen, Denmark; 2 Department of Neuroscience, University of Copenhagen, Blegdamsvej 3B, 2200 Copenhagen, Denmark; 3 Department of Biomedical Engineering, Lund University, Ole Römers Väg 3, 223 63 Lund, Sweden; 4 Department of Experimental Medical Science, Lund University, Sölvegatan 17, BMC-B11, 221 84 Lund, Sweden; 5 Wallenberg Center for Molecular Medicine, Lund University, Sölvegatan 17, BMC-B11, 221 84 Lund, Sweden

**Keywords:** Mass spectrometry, Dopamine progenitors, ELISA, Quality control, Biomarkers, Cell replacement therapy, Parkinson’s disease, Secretome, Extracellular vesicles

## Abstract

**Background:**

Ventral midbrain (VM) dopaminergic progenitor cells derived from human pluripotent stem cells have the potential to replace endogenously lost dopamine neurons and are currently in preclinical and clinical development for treatment of Parkinson’s Disease (PD). However, one main challenge in the quality control of the cells is that rostral and caudal VM progenitors are extremely similar transcriptionally though only the caudal VM cells give rise to dopaminergic (DA) neurons with functionality relevant for cell replacement in PD. Therefore, it is critical to develop assays which can rapidly and reliably discriminate rostral from caudal VM cells during clinical manufacturing.

**Methods:**

We performed shotgun proteomics on cell culture supernatants from rostral and caudal VM progenitor cells to search for novel secreted biomarkers specific to DA progenitors from the caudal VM. Key hits were validated by qRT-PCR and ELISA.

**Results:**

We identified and validated novel secreted markers enriched in caudal VM progenitor cultures (CPE, LGI1 and PDGFC), and found these markers to correlate strongly with the expression of *EN1*, which is a predictive marker for successful graft outcome in DA cell transplantation products. Other markers (CNTN2 and CORIN) were found to conversely be enriched in the non-dopaminergic rostral VM cultures. Key novel ELISA markers were further validated on supernatant samples from GMP-manufactured caudal VM batches.

**Conclusion:**

As a non-invasive in-process quality control test for predicting correctly patterned batches of caudal VM DA cells during clinical manufacturing, we propose a dual ELISA panel measuring LGI1/CORIN ratios around day 16 of differentiation.

**Supplementary Information:**

The online version contains supplementary material available at 10.1186/s13287-023-03580-5.

## Introduction

Parkinson’s disease (PD) is a common neurodegenerative movement disorder with a prevalence of 1% in the population above 60 years. PD involves the relatively selective loss of dopaminergic (DA) neurons within the *substantia nigra*, and it is the loss of this particular neuronal subtype which is the underlying cause of the main motoric symptoms in PD patients [1]. Based on this, DA cell replacement is a promising treatment strategy with the prospect of long-term symptomatic amelioration mediated by physiological DA release from transplanted DA neurons in the striatum. The feasibility and clinical efficacy of this approach has been demonstrated in studies using transplantation of fetal ventral midbrain (VM) tissue to the brains of PD patients [2–7]. Now, a new generation of cellular therapies derived through directed differentiation of human pluripotent stem cells (hPSCs) has emerged [8]. Here, hPSCs are differentiated in vitro specifically towards VM fates and then transplanted to the brain while still at the neural progenitor stage. The transplanted cells subsequently mature in the host brain to form functional DA neurons which can integrate and secrete dopamine to the surrounding host parenchyma.

To ensure safe, efficacious, and reproducible outcomes of stem cell-derived DA products, reliable and predictive quality control (QC) assays for correct DA progenitor fate must be applied. Intracellular proteins known to be expressed in VM DA progenitors, such as the transcription factors LMX1A, FOXA2 and OTX2 [9], are commonly used as surrogate markers for assessing the presence of DA precursor cells in transplanted cell populations. However, assessing intracellular marker expression by staining or RNA expression is invasive as it requires cellular fixation or lysis, and it is associated with significant sample processing time. Developing rapid and non-invasive QC measures which can identify correctly patterned VM DA fate from neural progenitor cells of non-DA fate is therefore of high value for producing cells for clinical use under Good Manufacturing Practice (GMP). Currently used QC assays further present the challenge that only caudal VM (cVM)-derived LMX1A/FOXA2/OTX2 triple-positive progenitors give rise to VM DA neurons whereas triple-positive cells of the rostral VM (rVM) produces other types of neurons, including glutamatergic neurons of the subthalamic nucleus [10, 11]. Hence, QC assays using LMX1A/FOXA2/OTX2 are unable to distinguish between cVM and rVM fates, and these markers alone—although necessary—are not sufficient to predict successful graft outcome upon transplantation [11]. We have previously shown that a reliable cVM marker, which can predict successful graft outcome from stem cell transplants, is the transcription factor EN1 [11]. However, a main hurdle in the field is that there are currently no commercially available antibodies for EN1 which work well on flow cytometry. Consequently, current clinical products rely on the use of FOXA2 and/or OTX2 by flow cytometry for assessment of VM cell purity in the final product [12, 13].

In this study, we searched for novel biomarkers secreted by hPSC-derived VM progenitor cells, with the specific quality of being able to distinguish correctly patterned cVM DA progenitors from the closely related non-DA rVM progenitors, as well as from neural progenitors of other brain regions. To identify secreted markers, we applied shotgun mass spectrometry-based (MS) proteomics on harvested medium from rVM and cVM cultures around day 16 of differentiation, which is the day at which progenitor cells are harvested for the purpose of clinical transplantation [14]. Top candidates from the MS analysis were validated by qRT-PCR and ELISA assay, and from this we identified several secreted markers which were present at significantly different levels in medium from rVM and cVM cultures. We further combined two of these markers to generate a dual ELISA panel which could robustly discriminate correctly patterned cVM cells for use in clinical transplantation therapy.

## Materials and methods

### Regionalized neural differentiation of hESCs

RC17 hESCs from Roslin Cells (Edinburgh, UK), normally karyotyped and mycoplasma-free, were maintained on Laminin 521 (Biolamina) -coated culture dishes (Sarstedt) in StemMACS iPS Brew XF medium (Miltenyi Biotec) and passaged with EDTA (0.5 mM) once weekly. The RC17 cell line used for this work is deposited in the UK Stem Cell Bank (https://nibsc.org/ukstemcellbank), and is registered in the online registry for human pluripotent stem cells hPSCreg (https://hpscreg.eu/, number RCe021-A).

The cells were differentiated towards progenitors of dorsal forebrain (dFB), ventral forebrain (vFB), dorsal midbrain (dMB), rostral ventral midbrain (rVM), caudal ventral midbrain (cVM), dorsal hindbrain (dHB), and ventral hindbrain (vHB) fates. For all conditions, media composition, coating, seeding densities and replating steps were followed until day 16 as previously described [11, 14]. All conditions received dual SMAD inhibition (SB431542 10 µM and Noggin 100 ng/ml) from day 0–9 of differentiation. Patterning into each of the different regions was obtained by differential addition of patterning factors CHIR99021 (referred to as CHIR), SHH-C24II (referred to as SHH) and FGF8b, all from Miltenyi Biotec, as follows: dFB (no additional factors added), vFB (SHH 300 ng/ml day 0–9), dMB (CHIR 0.7 µM day 0–9 + FGF8b 100 ng/ml day 4–16), rVM (CHIR 0.7 µM day 0–9 + SHH 300 ng/ml day 0–9), cVM (CHIR 0.7 µM day 0–9 + SHH 300 ng/ml day 0–9 + FGF8b 100 ng/ml day 9–16), dHB (CHIR 2 µM day 0–9) and vHB (CHIR 2 µM day 0–9 + SHH 300 ng/ml day 0–9). The basal medium used during differentiation of all regional fates consisted of DMEM/F12 (Invitrogen) mixed 1:1 with NeuroMedium, (Miltenyi), supplemented with 1% N2 supplement from day 0–11. From day 11–16, cells were kept in NeuroMedium (Miltenyi) supplemented with 2% NeuroBrew-21 (Miltenyi) as well as BDNF (20 ng/ml) and Ascorbic acid (0.2 mM). The cell culture medium was harvested from the cells on day 11, 14 and 16, and medium from all these three timepoints was pooled for vesicle preparation by centrifugation (Experiment 3b). For global secretome analysis (Experiments 1, 2 and 3a), bovine serum albumin originating from the B27 medium was first removed from the cultures by washing the cells three times in PBS on day 16. Subsequently, the cells were cultured in NeuroMedium with 0.2% N2 supplement for 24 h until medium harvest for MS analysis on day 17. This procedure allowed to remove BSA from the input medium for MS, thereby significantly lowering the background signal on the global secretome MS analysis.

### mRNA extraction and qRT-PCR

Samples were homogenized using a QiaShredder column and RNA was isolated using RNeasy Micro kit (both from Qiagen), running on a QiaCube instrument, according to the manufacturer’s procedures. Reverse transcription was performed with random hexamer primers and Maxima First Strand cDNA Synthesis Kit (Thermo Scientific) using up to 1 μg of RNA from each sample. The complementary DNA was pipetted onto a 384-well plate, together with SYBR green Mastermix (Roche Life Sciences) and primers using an automated liquid handler (I.DOT One, Dispendix). Samples were analyzed by real-time quantitative PCR on a LightCycler 480 instrument (Roche Life Sciences) using a two-step protocol with a 60 °C annealing/elongation step, for 40 cycles (Ct calculations capped at 35). All qRT-PCR samples were run in technical duplicates, and the averaged Ct values were used for calculations. Data are represented using the ΔΔCt method. For each gene and samples, the fold change was calculated as the average fold change relative to undifferentiated hESCs, based on two different housekeeping genes (*ACTB* and *GAPDH*). List of primers used, and respective sequence is provided in Additional file 1: Table S1.

### Sample preparation for whole supernatant (Global Secretome) for MS

**Fig. 1:**
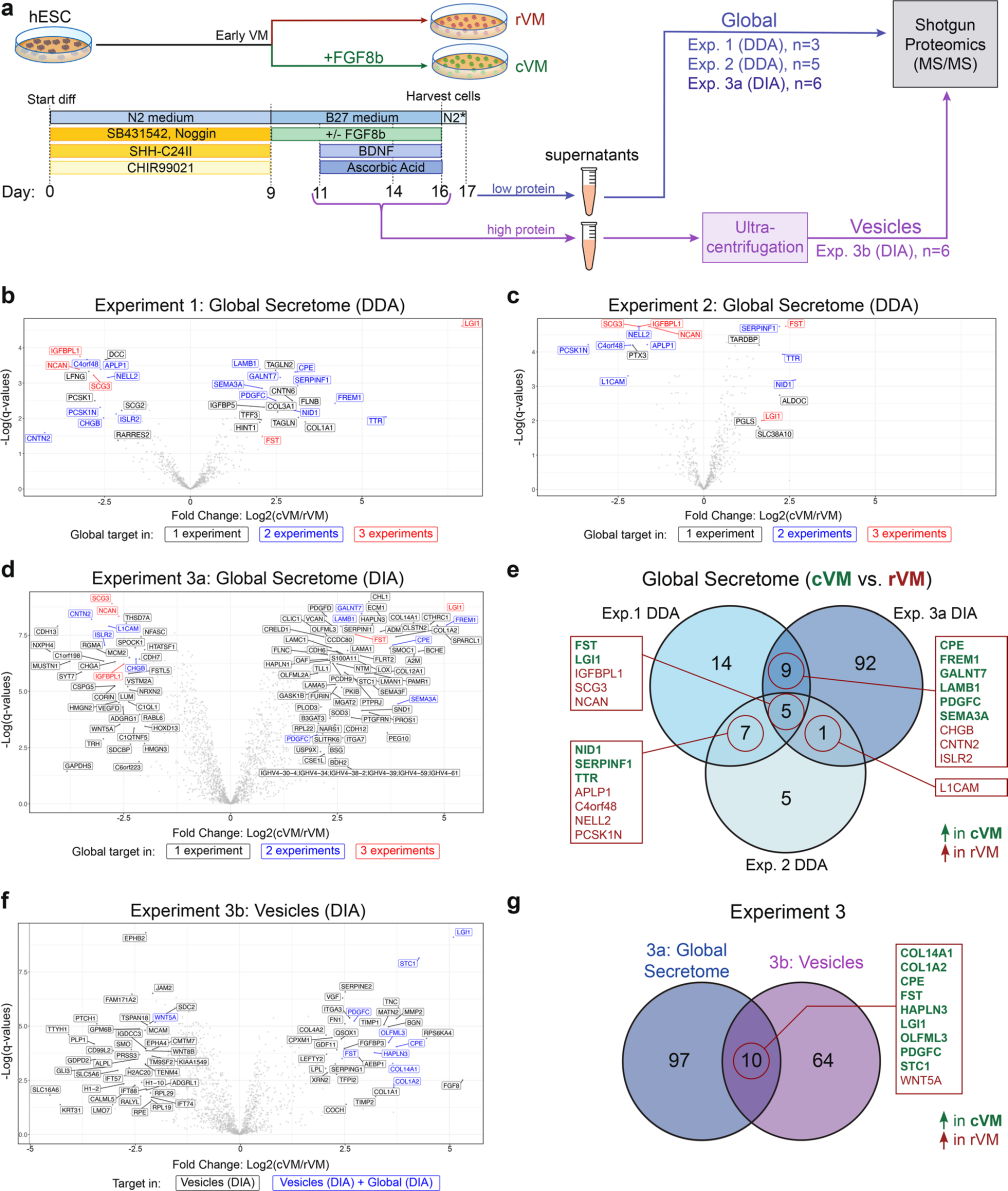
Shotgun proteomics-based protein discovery of proteins differentially enriched in the supernatant of hESC-derived ventral midbrain (VM) cell culture. **a** Schematics of the experimental set-up for hESC cell differentiation supernatant protein discovery. For each experiment, the supernatant of *n* = 3–6 biological replicates of rVM and cVM samples was collected. The differentiation procedure for the VM regions only differs with the addition of FGF8b at day 9 for the cVM condition. Three different MS/MS experiments were run for global secretome analysis. In addition, vesicle collection by ultra-centrifugation was performed in the third experiment. N2*: 0.2% N2 supplement. **b–d** Volcano plots for the proteins differentially detected between the rVM and cVM supernatants, in Global Secretome experiments: Experiment 1 DDA (**b**), Experiment 2 DDA (**c**) and Experiment 3a DIA (**d**). Targets found in all 3 Global experiments are Red-labelled, while Blue-labelled targets were shared between 2 of the Global experiments. **e** Venn diagram showing the overlapping hits obtained from the three different Global Secretome experiments. Targets enriched in cVM samples are highlighted in green, and targets in rVM in red. **f** Volcano plots for Experiment 3b Vesicle DIA, showing the proteins differentially detected between rVM and cVM. Blue-labelled targets were found shared in both Experiment 3a Global Secretome DIA and Experiment 3b Vesicle DIA. **g** Venn diagram showing the overlapping hits between the Global Secretome and Vesicles in Experiment 3. Targets enriched in cVM samples are highlighted in green, and targets in rVM in red

Media samples from VM cultures harvested at day 17 after 24 h of culturing in low protein-content media containing 0.2% N2 supplement (Fig. 1a, Experiment 1, *n* = 3 biological replicates, Experiment 2, n = 5 biological replicates, Experiment 3a, *n* = 6 biological replicates) were prepared for mass spectrometry using in-solution digestion. In this study, a “biological replicate” was defined as a sample obtained from a separate round of differentiation, i.e. a new experiment where the entire differentiation procedure was repeated with a new passage of pluripotent, undifferentiated hESCs. Proteins were denatured with 8 M Urea (50 mM Ambic) and reduced with 10 mM (50 mM AmBic) Dithiothreitol (DTT) at 56 °C for 1 h with 900 rpm shaking. Subsequently, samples were alkylated with 20 mM (50 mM AmBic) Iodoacetamide (IAA) in darkness for 30 min at room temperature. Ethanol was added to all samples with a ratio 1:9 (v/v, sample:ethanol) for protein precipitation and incubated over night at − 20 °C. After precipitation, samples were centrifuged at 12,000 rpm × 15 min at 4 °C and ethanol was removed with a pipette. Protein pellets were dried in a concentrator to remove any remaining trace of ethanol, followed by pellet dissolution in 100 µl 50 mM AmBic. For protein digestion, 2 µg Trypsin with a ratio 1:50 (w/w, Trypsin:sample) was added to each sample followed by incubation at 37 °C for 17 h with shaking (350 rpm). Protein digestion was stopped by reducing pH to 4 with Formic acid (v/v 10% in AmBic). iRT peptides (Biognosys AG) were added to each sample in a ratio 1:10 (v/v iRT:sample). Samples were then dried in a concentrator and stored at − 80 °C.

### Preparation of vesicle-enriched samples for MS

To enrich for secreted vesicles, media samples harvested at day 11, day 14 and day 16 (see Fig. 1a, Experiment 3b, *n* = 6 biological replicates) were run in a differential centrifugation protocol in the following order: 300g× 10 min at 4 °C, 2000g× 10 min at 4 °C and 10,000g× 30 min at 4 °C. In between each centrifugation step, the supernatant was transferred to new tubes. Media samples from the same cultures were pooled and transferred to ultracentrifugation tubes. Samples were ultra-centrifuged at 100,000*g*× 70 min at 4 °C. The supernatant was discarded and 12 ml 50 mM AmBic was added to the top of each tube to wash the pellet, followed by another ultra-centrifugation step at 100,000g× 70 min at 4 °C. After centrifugation, the top 11 ml of media was discarded while the remaining 1 ml volume was mixed with a pipette to dissolve the vesicle pellet. The 1 ml sample was then transferred to new tubes for MS sample preparation. Sample volumes were reduced to 100 µl using a concentrator, followed by the addition of 50 µl RIPA buffer for vesicle lysis and protein denaturation. To further improve lysis, samples were placed in a Bioruptor 300 sonication system (Diagenode) and run for 50 cycles (High Power 15s/OFF 15s) at 4 °C. After lysis, proteins in the samples were reduced, alkylated and precipitated according to the method for the whole supernatant samples as described above. After precipitation, samples were centrifuged at 14,000 rpm × 15 min at 4 °C and the supernatant was discarded. Samples where further dried in a concentrator to remove any trace of ethanol. To dissolve the pellet, 50 µl AmBic (100 mM) was added to each sample. In order to remove glycosylations on Asparagine residues, 1.5 µl PNGase F (Promega) was added to each sample and incubated for 18 h with little shaking. For protein digestion 1.4 µg Trypsin was added to each sample with a ratio 1:50 (w/w, Trypsin:sample) and incubated at 37 °C for 22 h with shaking (350 rpm). Protein digestion was stopped with 10 µl Formic acid (v/v 10% in AmBic). Samples were dried in a concentrator and stored in − 80 °C.

### Data-dependent acquisition MS runs (DDA)

Supernatant samples from cVM and rVM (Experiment 1) were run in DDA mode on a Q Exactive Plus (Thermo Fisher Scientific) to be used for subsequent global DDA analysis. An EASY-nLC 1000 ultra-high-performance liquid chromatography system (Thermo Fisher Scientific) was connected to the MS instrument. Peptide separation was performed on an EASY-Spray column (ES802, Thermo Fisher Scientific) by running a linear acetonitrile gradient going from 5 to 30% solvent B (0.1% formic acid in acetonitrile) for 90 min. As solvent A, 0.1% formic acid was used. MS1 spectra were acquired in profile mode with a resolution of 70,000. In each cycle, the top 15 most intense precursor were selected in MS1 for fragmentation, but with a dynamic exclusion time of 20 s. Acquired MS2 spectra were centroided, with a resolution of 17,500. Normalized collision energy for fragmentation (NCE) was set to 30. Scan range in MS1 and MS2 was set to 400–1600 m/z and 200–2000 m/z respectively. Automatic gain control (AGC) target was set to 1e6 in both MS1 and MS2. Maximum ion injection time (IT) was set to 100 ms in MS1, and 60 ms in MS2.

In order to build sample-specific spectral libraries for later DIA analyses (Experiment 3), supernatant samples from cVM and rVM (global DIA and vesicles DIA dataset), were run on a Q Exactive HF-X (Thermo Fisher Scientific) in DDA mode. Connected to the MS instrument was an EASY-nLC 1200 ultrahigh-performance liquid chromatography system (Thermo Fisher Scientific). An EASY-Spray column (ES803, Thermo Fisher Scientific) separated peptides in a non-linear acetonitrile gradient for 2 h (solvent B | 1% to 7%:8 min, 7% to 12%:15 min, 12% to 27%:65 min, 27% to 32%:15 min, 32% to 37%:9 min, 37% to 52%:8 min, 52% to 90%: 2 min). MS1 spectra recorded in profile mode had a resolution of 120 000. The top 20 most abundant precursors were chosen for fragmentation in each cycle, and the dynamic exclusion time was set to 15s. Centroided MS2 spectra were acquired at a resolution of 15,000, with NCE = 27. Scan ranges were set to 350–1650 m/z in MS1, and 200–2000 m/z in MS2 respectively. The AGC target was set to 3e6 in MS1, and 1e5 in MS2. The maximum IT was set to 20 ms in MS1, while it was set to 20 ms in MS2.

### Data-independent MS acquisition (DIA)

Samples for all DIA analyses were acquired on a Q Exactive HF-X mass spectrometer (Thermo Fisher Scientific), using the same liquid chromatography (LC) system and gradient settings as for the global DDA runs to build spectral libraries. For data-independent acquisition (DIA), the instrument method was set to acquire a full MS1 scan (resolution 120,000, scan range: 350–1650 m/z) in profile mode, followed by 44 variable MS2 windows (resolution 30,000) with the following ranges: 350–371, 370–387, 386–403, 402–416, 415–427, 426–439, 438–451, 450–462, 461–472, 471–483, 482–494, 493–505, 504–515, 514–525, 524–537, 536–548, 547–557, 556–568, 567–580, 579–591, 590–603, 602–614, 613–626, 625–638, 637–651, 650–664, 663–677, 676–690, 689–704, 703–719, 718–735, 734–753, 752–771, 770–790, 789–811, 810–832, 831–857, 856–884, 883–916, 915–955, 954–997, 996–1057, 1056–1135 and 1134–1650 m/z. A stepped NCE was used for fragmentation (NCE = 25.5, 27, 30). AGC targets were set to 3e6 in both MS1 and MS2. Maximum IT was set to 60 ms in MS1 and ‘auto’ in MS2.

For later spectral library building, pooled supernatant samples (Global) and vesicle samples respectively, were run with gas-phase fractionated (GPF) DIA methods. For the pooled supernatant samples, there were 6 methods with DIA windows covering different MS1 ranges (400–500 m/z, 500–600 m/z, 600–700 m/z, 700–800 m/z, 800–900 m/z, 900–1000 m/z). Centroided MS1 and MS2 spectra were recorded with a resolution of 30 000. For the pooled vesicles samples, there were 10 GPF-DIA methods with DIA windows covering 10 different MS1 ranges respectively (300–400 m/z, 400–500 m/z, 500–600 m/z, 600–700 m/z, 700–800 m/z, 800–900 m/z, 900–1000 m/z, 1000–1100 m/z, 1100–1200 m/z, 1200–1650 m/z).

For each GPF-DIA method, a set of 51 overlapping DIA windows with a fixed window size of 4 m/z were acquired to cover the full MS1 ranges. The only exception was the GPF-DIA method for the 1200–1650 m/z range, having a fixed window size of 18 m/z. The AGC target was set to 3e6 in MS1, and 1e6 in MS2.

### DDA-based spectral library generation

DDA MS raw files belonging to Experiment 3 (Global and Vesicles) were imported into Fragpipe v.16.1-build5 (https://github.com/Nesvilab/FragPipe). As database, the human proteome FASTA file was used (UP000005640, Uniprot/Swissprot release 21_03) with decoys appended (reversed target sequences). To build the spectral library, the default ‘SpecLib’ workflow was loaded and the default settings for all tools were used. In this workflow, the database search engine MSFragger v3.3 [15] was employed to identify MS/MS spectra, followed by Percolator [16] for confidence estimation. Protein grouping and post processing was performed using ProteinProphet [17] and Philosopher [18] followed by spectral library building with EasyPQP (https://github.com/grosenberger/easypqp).

#### DIA-based spectral library generation

DIA raw files were loaded into DIA-NN v.1.8 [19] to build a wide-window DIA spectral library for the global dataset and the vesicle dataset respectively. Confidently identified spectra (*q*-value ≤ 0.01) were extracted from each DIA file to be included in the final library. Narrow-window libraries were also built in DIA-NN for both datasets, using acquired GPF-DIA runs. Similarly, wide-window DIA spectral libraries were built for both datasets in Fragpipe v.16-build5 using the existing workflow ‘MSFragger-DIA-wide-window-SpecLib’. Also, narrow-window spectral libraries were built with the workflow option: ‘MSFragger-DIA-narrow-window-SpecLib’ using default settings. For all spectral libraries the canonical human proteome FASTA database was used (UP000005640, Uniprot/Swissprot release 21_03).

### Super spectral library generation

In total, ten different spectral libraries were built for Experiment 3, five for each of the analyses, Global and Vesicles. As different library building strategies resulted in slightly different targets, the libraries were imported into R (v.4.2.1) and combined into non-redundant super spectral libraries, one for each dataset, using a custom R script.

### Data analysis of global DDA runs

Raw DDA files acquired by DDA on the Q Exactive Plus were loaded into MaxQuant v.1.6.1.0 [20–22] for label-free quantification of proteins. DDA MS files were put in different parameter groups based on their Experiment (1 or 2) to ensure batch-specific normalization and quantification with the MaxLFQ algorithm [23]. Identification settings used the default false-discovery rate of 1% on protein, peptide and peptide-spectral-match level. As FASTA database, the human canonical proteome was used (UP000005640, Uniprot/Swissprot release 21_03). Match-between-runs to transfer identifications between runs was enabled. Carbamidomethylation on Cystein (UniMod:4) was set as fixed modification and variable modifications were oxidation on Methionine (Unimod:35) and acetylation on protein N-terminal (UniMod:1). For label-free quantification, it was required that at least one peptide was identified from MS/MS for pairwise comparisons. The minimum LFQ peptide ratio was set to 1, in order to allow more low-abundant proteins to be quantified.

### Data analysis of DIA runs

Acquired DIA raw files acquired on the Q Exactive HF-X were searched against their respective super spectral library in DIA-NN v.1.8 [19]. The quantification strategy was set to ‘Robust LC (high accuracy)’ while cross run normalization was set to RT-dependent (default). Based on the median recommended MS1 accuracies reported by DIA-NN for each run, the MS1 accuracy was set to 7.96 ppm for the Global DIA dataset (Experiment 3a) while being set to 8.48 ppm for the Vesicles dataset (Experiment 3b). MS2 accuracies were automatically set by DIA-NN to 20 ppm for both analyses. Relaxed protein inference was enabled in DIA-NN to avoid the assignment of the same protein to more than one group during protein inference. The human proteome FASTA file (UP000005640, Uniprot/Swissprot release 21_03) was used for annotations in DIA-NN.

### ELISA

Supernatant samples were collected from the differentiating cells at the day 11 and 16 in their regular B27 medium, and immediately frozen. ELISA kits for the targets proteins were used in according to the manufacturer’s instructions: CNTN2, CORIN, FST, PDGFC, SERPINF1, TFF3 (all from R&D Systems), CPE (Nordic Biosite), LGI1 (Cusabio) (see Additional file 1: Table S2). Before analysis, each supernatants sample was centrifuged at > 10.000 rpm for 10 min to remove cell debris. Initial tests were performed to ascertain dilution factors for the various proteins and samples, although some measurements were above or below the detection limit. Sample measurements above detection limit were excluded. Samples assayed at 1:1 dilution and with measurements below the detection limit were attributed the Minimum Detectable Dose according to the manufacturer’s information, or, in the absence, the minimum calculatable value using the respective dilution curve and 4-PL curve fit. The measured protein concentration values were then normalized to the cell count in the respective well, yielding pg.ml^−1^.10^–6^ cells.

#### Statistical analysis of ELISA and qRT-PCR data

All ELISA and qRT-PCR data was managed in Excel and statistically analyzed using GraphPad Prism 9 software, *P* < 0.05 was considered significant. For multi-regional comparisons, one-way analysis of variance (ANOVA) was performed followed by a Sidak multiple comparison test between the rVM and cVM and remaining regions. All datasets were tested for their normal and Log-Normal distribution (Shapiro– Wilk and Kolmogorov–Smirnov) and homoscedasticity (Brown–Forsythe) before ANOVA. Alternatively, a non-parametric Kruskal–Wallis analysis was conducted instead, followed by a Dunn’s multiple comparison test. All multiple comparison tests were corrected using statistical hypothesis testing.

For pairwise comparison between rVM and cVM, a two-tailed unpaired t-Test was performed, or in case the datasets and the Log-transformed datasets lacked a Gaussian distribution or showed significantly different variances, a Mann–Whitney test was performed instead.

For calculating the correlation between the *EN1* mRNA expression and the ELISA-assayed Protein levels, a two-tailed Spearman correlation was performed on the Log–Log data. A straight, non-linear, least squares regression was fitted to the Log–Log data, computing the 95% confidence interval.

#### Statistical analysis of DDA and DIA analyses

Result files from the Global analysis and the Vesicles analysis were imported into R for processing and differential expression analysis. The protein groups table (proteinGroups.txt) from the MaxQuant search was filtered to not contain decoys nor entries only identified by site. A quantitative matrix was extracted by selecting the ‘LFQ intensity’-columns from the table, and the quantitative values were subsequently log2-transformed. Imputation was applied to the matrices using the R package imputeLCMD [24] v.2.0, where the K-nearest neighbors algorithm impute values missing at random, while the ‘MinProb’-algorithm was used to impute values missing not at random. Differential expression analysis was performed by running a moderated t-test using the R package DEqMS [25] v.1.8.0 to compare samples belonging to cVM with those in rVM. For each test, DEqMS reported a fold change, a spectra count adjusted p-value (sca.*P*.value) and a spectra count false discovery rate-adjusted p-value (sca.adj.pval) = *q*-value, Benjamini–Hochberg method [26]. Tests were regarded as significant if the *q*-value was ≤ 0.05 and the fold change was larger than ± 2. For Experiment 2, Global DDA, a cut-off fold change of ± 1.5 was applied due to poorer resolution in this experiment.

Output reports from DIA-NN, for the Global analysis and Vesicles analysis, were imported into R for downstream processing. Reports were filtered to only contain confidently identified entries (Global precursor *q*-value ≤ 0.01, Global protein group *q*-value ≤ 0.01). Quantitative protein groups matrices were computed with the MaxLFQ [23] algorithm, implemented in the R package ‘diann’ v.1.0.1 (https://github.com/vdemichev/diann-rpackage). Following log2-transformation, the matrices were filtered to only contain protein groups having at least 60% quantitative values evenly distributed among samples in both conditions (cVM or rVM), or at least 50% quantitative values given that all were present in one group only. Retained protein groups were then imputed using the ‘MinProb’ algorithm described above (see global DDA analysis). Similarly to the global DDA analysis, DEqMS [25] v.1.8.0 was used to perform differential expression analysis between samples in the cVM condition and the rVM condition.

#### GO-term enrichment analysis

A GO-term enrichment analysis for cellular components between the Global DIA dataset and the Vesicles DIA dataset was performed in R (v.4.2.1) with the package Clusterprofiler [27] v.3.18.1. To find enriched GO-terms for cellular components in the Global DIA dataset, the enrichGo function was used to query gene names for identified proteins in the Global DIA dataset against all identified gene names (Global DIA + Vesicles DIA). Inversely, all gene names in the Vesicles DIA dataset were queried against all identified gene names to find enriched cellular component GO-terms in the Vesicles dataset. Only significant results were considered (*q*-value ≤ 0.05, Benjamini–Hochberg FDR estimation [26]).

## Results

### Identifying secreted biomarkers from ventral midbrain progenitor cells through shotgun proteomics

To identify relevant secreted protein candidates from cVM DA progenitor cultures, shotgun proteomics was used to analyze whole supernatant collected from cultures of hESC-derived VM cells. For this purpose, we applied a clinical grade cell line (RC17) and a differentiation protocol adapted to Good Manufacturing Practice (GMP) for producing rostral and caudal VM progenitor cells (rVM and cVM, respectively) [14], thus performing the analysis on clinically relevant cell populations. Thereby, the differentiated cVM cell populations used in this study are equivalent to cells in the STEM-PD product which is currently in clinical trial for treatment of  Parkinson’s Disease [8, 13, 28], and all rVM and cVM batches used in this study were assessed for correct differentiation using a qRT-PCR panel designed specifically for quality control of rVM and cVM batches [14]. The global secretome was analyzed in medium which was harvested from the cells around the time of transplantation (i.e., collected from day 16 to day 17 of differentiation). However, to reduce background signals of Albumin, Serotransferrin and Insulin from the basic B27-supplement-containing cell medium, cell cultures were washed three times in PBS on day 16 of differentiation, and medium was changed to a low-protein content media with 0.2% N2 supplement, which was harvested 24 h later (day 17, see Materials and Methods and Fig. 1a). In two initial experiments (Experiment 1, *n* = 3 biological replicates and Experiment 2, *n* = 5 biological replicates), Data-dependent acquisition (DDA) with label-free quantification (LFQ) was used to measure the relative protein abundances between rVM and cVM culture supernatants (Fig. 1b, c). To allow for a deeper protein quantification, screening less abundant targets, a third experiment (Experiment 3a, *n* = 6 biological replicates) was carried out where quantification was obtained through Data-independent acquisition (DIA) followed by LFQ (Fig. 1d). Differential expression analysis showed several upregulated proteins in the cVM supernatant that were shared in at least 2 out of 3 experiments, such as LGI1 (Leucine-rich glioma-inactivated protein 1), FREM1 (FRAS1-related extracellular matrix protein 1), CPE (Carboxypeptidase E) and SERPINF1 (Serpin family F member 1). Likewise, several protein candidates were found to be enriched in the rVM condition, such as CNTN2 (Contactin-2), PCSK1N (Proprotein convertase subtilisin/kexin type 1 inhibitor) and NCAN (Neurocan) (Fig. 1e, for full dataset see Additional file 2: Table S4).

### Comparing the global secretome with the proteome of vesicles

In the latest years there has been a rise in awareness to the role of extracellular vesicles in intercellular communication [29] as well as their potential as an accessible biological source to identify biomarkers by proteomic analysis [30, 31]. However, many vesicle-bound proteins may be lowly abundant and therefore difficult to detect by MS in the global secretome samples, although they could still constitute feasible targets for sensitive ELISA analysis. Therefore, to ensure the detection of differentially expressed lowly abundant vesicle-associated proteins, we performed shotgun proteomics on enriched vesicles sourced from the same cell batches as were analyzed in Experiment 3a. To this end, as the vesicle proteomics required rather large volumes of medium to capture sufficient vesicular material, pooled supernatant samples collected from rVM and cVM cultures in regular B27 medium at day 11, 14 and 16 (i.e. the days where medium change is performed on the cells) were enriched for their extracellular vesicle content by ultra-centrifugation and analyzed using DIA (Experiment 3b, hereafter termed “Vesicles”, Fig. 1a). Similarly, LFQ followed by differential expression analysis between the rVM and the cVM samples was performed (Fig. 1f), adding a dataset of 74 differentially enriched protein targets, including STC1 (Stanniocalcin 1), OLFML3 (Olfactomedin-like protein 3) and PDGFC (Platelet-derived growth factor C), which were found to be upregulated in cVM vesicle samples. While the majority of the protein targets were unique to our vesicle-enriched samples, i.e. not found in any of the whole supernatant datasets, 4 cVM enriched targets proteins were shared with at least 2 other datasets: CPE, FST (Follistatin), LGI1 and PDGFC (Fig. 1g).

Gene ontology analysis confirmed the differential origin of the analyzed samples, with the global samples showing an enrichment for proteins of extracellular matrix as well as proteins of the secretory lumen of the endoplasmic reticulum, while the vesicle samples were enriched for membrane and ribosomal proteins (Additional file 1: Fig. S1a). Furthermore, several protein markers characteristic of extracellular vesicles, such as ALIX (*PDCD612P*), TSG101, CD63, CD81, CD47 and VPS4B [32, 33] were almost exclusively detected in our vesicle samples (Additional file 1: Fig. S1b) in accordance with the guideline for minimal information for studies in extracellular vesicles [34]. None of these baseline proteins were differentially enriched in either rostral or caudal VM samples (Additional file 1: Table S3).

From the resulting MS datasets, we next selected a list of potential candidate proteins for validation by qRT-PCR and ELISA, choosing the candidates from the following characteristics: (a) confirmed identification as a differentially expressed target in two or more datasets, (b) high fold-change difference between the two VM regions and (c) the availability of a reliable commercial source of ELISA assays for detection of the proteins. Based on these parameters, we selected 6 differentially expressed secreted protein candidates for ELISA validation: FST, LGI1 (present in all 4 datasets), CPE, PDGFC (present in 3 datasets) and SERPINF1 (present in 2 datasets) as candidates enriched in cVM samples, and CNTN2, the most enriched rVM marker present in more than one dataset. CORIN was also added for validation not only because it was found to be enriched in the DIA global secretome analysis (Table 1), but also because this protein was previously found to be enriched on the cell surface of rVM progenitor cells compared to cVM progenitors [11]. All 7 candidates showed robust peptide detection as assessed by profile plots (Additional file 1: Fig. S1c, d). Furthermore, we included TFF3 (Trefoil factor 3) on the validation list, as this factor was previously identified as an enriched marker in DA VM progenitor cells by another group [35], though it was only barely identified in our first DDA analysis (see Table 1).

**Table 1:** Differential detection of candidate protein markers in all datasets

	Experiment 1, Global DDA		Experiment 2, Global DDA		Experiment 3a: Global DIA		Experiment 3b: Vesicles DIA	
Protein	Fold change	*q*-value	Fold change	*q*-value	Fold change	*q*-value	Fold change	*q*-value
CNTN2	***− 4.25***	***0.025***	− 1.29	6.33E−05	***− 3.25***	***6.19E−09***	-0.98	1.23E−03
CPE	**3.05**	**4.85E−04**	0.69	0.019	**3.80**	**8.62E−08**	**3.74**	**8.96E−05**
FST	**2.10**	**0.033**	**2.39**	**1.81E−05**	**2.93**	**2.91E−08**	**2.44**	**3.15E−04**
LGI1	**7.91**	**2.41E−05**	**1.69**	**9.90E−03**	**5.43**	**1.27E−09**	**5.10**	**7.84E−10**
PDGFC	**2.48**	**3.14E−03**	0.48	0.141	**2.02**	**8.53E−04**	**2.73**	**5.90E−06**
SERPINF1	**3.12**	**1.19E−03**	**2.21**	**1.81E−05**	0.40	0.088	1.49	0.121
CORIN	ND	ND	ND	ND	− ***2.71***	***5.76E−06***	-1.65	0.011
TFF3	**2.07**	**0.011**	ND	ND	ND	ND	ND	ND

Differential detection of the supernatant target proteins selected for further validation, from all MS analyses and experiments. Significant differential expression is indicated in bold font, and is defined as fold changes larger than ± 2 (± 1.5 for experiment 2—Global DDA) and *q*-value < 0.05 (*q*-value = sca.adj.pval). Bold is upregulated in cVM and bold italics is upregulated in rVM. ND: not detected. Full dataset is provided in Additional file 2: Table S4

### Validating expression profiles of secreted cVM candidates in a new set of samples

**Fig. 2:**
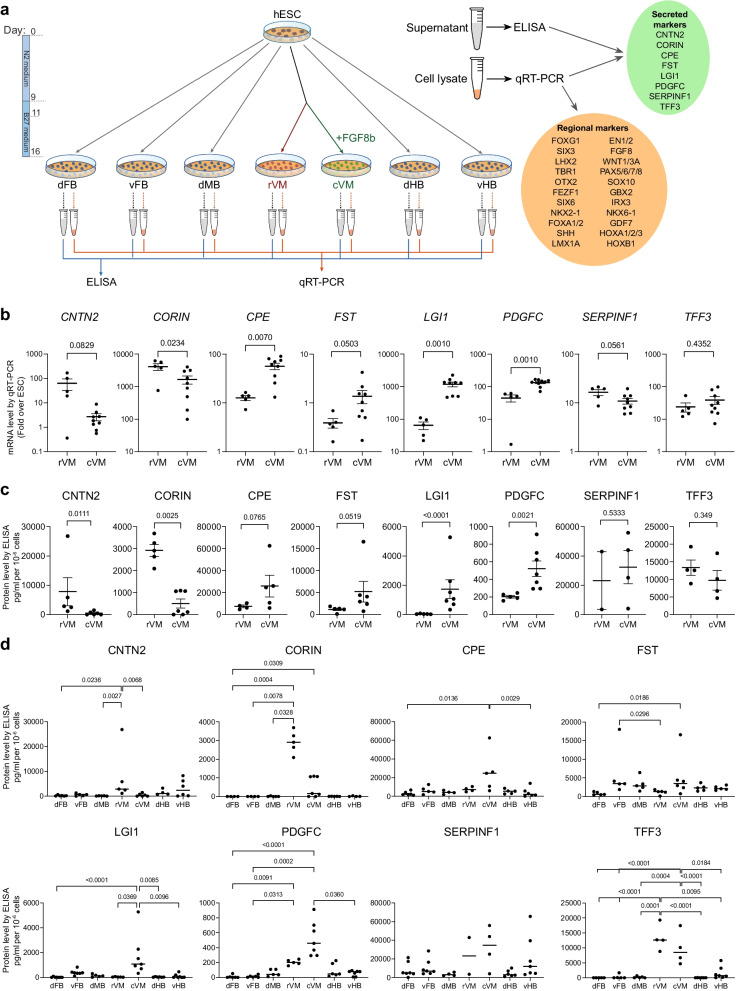
Supernatant analysis by ELISA, and qRT-PCR analysis of the rVM and cVM samples. **a** Schematics of the experimental set-up for obtaining supernatant and RNA samples from hESC differentiated to different neural locations: dorsal Forebrain (dFB), ventral Forebrain (vFB), dorsal Midbrain (dMB), rostral ventral Midbrain (rVM), caudal ventral Midbrain (cVM), dorsal Hindbrain (dHB), ventral Hindbrain (vHB). **b** Quantitative reverse-transcription PCR analysis of rVM and cVM hESC-derived cells for the expression of the selected target proteins. **c** Quantification of supernatant proteins at day 16 of differentiation in cVM and cVM cultures by ELISA. **d** Quantification of supernatant proteins by ELISA, in a panel of hESC-derived cells differentiated to various neural tube regions, at day 16 of differentiation

To further assess the discriminative potential of the selected candidate markers in a new set of differentiated neural progenitor samples, we performed quantitative reverse-transcription PCR (qRT-PCR) analysis for the expression of the respective genes in rVM and cVM cultures on day 16 of differentiation. To this aim, we created a new set of samples obtained from hESC differentiated towards rVM and cVM as well as towards other neural tube regions for comparison (Fig. 2a). In line with the MS data, we observed that transcription of *CORIN* was significantly upregulated in the rVM samples, while *CPE*, *LGI1* and *PDGFC* expression was increased in the cVM samples (Fig. 2b). Though not statistically significant, *CNTN2* and *SERPINF1* appeared to be increased in rVM and *FST* elevated in cVM. On the other hand, *TFF3* expression was indistinguishable between the two regional VM samples. We then performed ELISA on the supernatant of the rVM and cVM samples, this time using direct sampling of B27 culture medium from day 16 of differentiation to mimic an in-process QC assay performed on the day of cell harvest for cryopreservation and subsequent clinical transplantation. The ELISA confirmed that CNTN2 and CORIN were elevated in the supernatant from the rVM cultures, while CPE, LGI1 and PDGFC were enriched in the cVM cultures (Fig. 2c). In line with the transcriptional data, FST, SERPINF1 and TFF3 showed no statistically significant difference between the VM regions. SERPINF1 ELISA analysis was particularly fraught by a high spread in protein concentration, despite preliminary dilution testing, resulting frequently (over 30%) in values above the detection limit.

We next proceeded to assess the specificity of these markers in VM cultures compared to neural progenitors of other regional fates. To this aim, hESCs were differentiated to other neural tube regions (dorsal Forebrain, dFB; ventral Forebrain, vFB, dorsal Midbrain, dMB, dorsal Hindbrain, dHB; and ventral Hindbrain, vHB), and the cultures were verified for correct regional fates by qRT-PCR using a panel of regional neural tube markers [36] (Additional file 1: Fig. S2a). By performing ELISA on the supernatant samples, we could generally observe elevated protein levels of the selected markers in the VM samples in comparison with the other neural regions. In particular, CORIN showed a clear specificity to the rVM whereas PDGFC was specific to the cVM, compared to all other neural regions tested. TFF3 depicted a strong enrichment for both VM regions in comparison to all other neural regions (Fig. 2d). Our data however showed that although TFF3 was a highly specific secreted marker of the VM, it could not discriminate between rostral and caudal VM samples.

### Designing a dual ELISA panel for discriminating cVM samples with predictive markers of efficacy

**Fig. 3:**
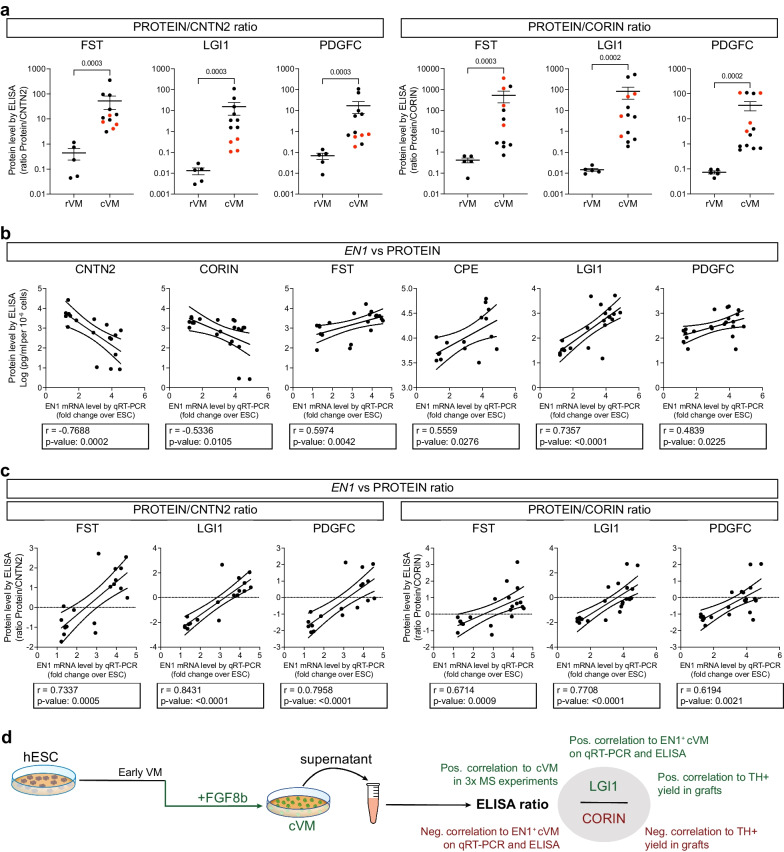
Correlation of supernatant and qRT-PCR analysis on VM samples. **a** Protein content ratio between the cVM markers (FST, LGI1, PDGFC) and the rVM markers (CORIN and CNTN2) in rVM and cVM supernatants at day 16 of differentiation. Red dots represent the analysis of 4 supernatant samples obtained from clinical-grade batches of cVM DA-progenitors. **b** Spearman Correlation analysis between the mRNA expression of the cVM DA-progenitor marker EN1 and the protein supernatant content in all VM samples. The non-linear regression of the Log–Log data is presented, with the 95% CI (dotted lines). **c** Spearman Correlation analysis between the mRNA expression of the cVM DA-progenitor marker EN1 and the selected cVM/rVM protein marker ratios at day 16 of differentiation. The non-linear regression of the Log–Log data is presented, with the 95% CI (dotted lines). **d** Proposed optimal ELISA read-out for predicting efficient patterning of cVM batches, consisting of a dual ELISA panel measuring LGI1/CORIN ratios around day 16 of differentiation

Given the observations above, we next asked if we could apply some of the identified markers as a potential non-invasive, quality control method to distinguish a successful hESC differentiation towards *bona fide* cVM DA-progenitors from an unsuccessful differentiation towards the non-dopaminergic rVM. We first investigated whether supernatant harvested at an earlier time point could predict the outcome of the VM cell fates on day 16. However, the ELISA analysis on day 11 supernatants showed low levels for all selected proteins, with no significant difference between rVM and cVM samples (Additional file 1: Fig. S2b). We therefore focused on developing a QC assay for assessment of the cultures at day 16 and hypothesized that combining the measurements of two secreted markers could provide an optimized non-invasive assay with higher reliability and without the need of a normalizing to cell count. Based on our day 16 results, we calculated the ratio between positive markers for cVM (CPE, FST, LGI1, PDGFC) and either a VM specific marker (TFF3) or a marker enriched in rVM samples (CNTN2 or CORIN). We observed that TFF3 worked poorly as a counterbalance marker due to its variable results, including analyses over the detection limit, despite previous dilution testing (Additional file 1: Fig. S3a). On the other hand, ratios of the positive cVM markers CPE, FST, LGI1 and PDGFC over CORIN or CNTN2 showed significant discrimination between rVM and cVM (Additional file 1: Fig. S3b, c). To substantiate our findings in a clinically relevant context, we subjected 4 supernatant samples from clinical batches of day 16 cVM-DA progenitor cells (STEM-PD product, manufactured under GMP conditions) to the same ELISA panel. We confirmed that the protein ratios of these GMP-produced samples fell in line with the other correctly specified research-grade cVM samples (GMP samples marked in red in Fig. 3a).

To further investigate the predictive value of these secreted markers, we assessed the relationship between the ELISA assayed proteins and the transcriptional expression of *EN1*, which is a highly relevant progenitor cell marker predictive of successful graft outcome with *bona fide* midbrain DA neurons required for PD cell therapy [11, 14, 37]. By performing a Spearman correlation analysis between the *EN1* mRNA expression levels in VM cells at day 16 and the secreted QC candidate proteins in the supernatant, we found that CPE, FST, LGI1 and PDGFC correlated positively with *EN1* expression levels, whereas CNTN2 and CORIN correlated negatively, as expected from the rVM versus cVM enrichment profile for these markers, respectively (Fig. 3b). In contrast, SERPINF1 and TFF3 levels showed no correlation to *EN1* expression on day 16 (data not shown). Importantly, the combined protein ratios were also positively correlated with high *EN1* expression (Fig. 3c), thereby further emphasizing the predictive value of this proposed dual ELISA QC assay for GMP manufacturing. Specifically, the LGI1 ratios presented the most stringent and strongest positive correlation with *EN1* expression. To assess the potential value of the candidate secreted markers in predicting in vivo graft outcome, we revisited a previously performed RNAseq-in vivo efficacy correlation study from our lab, involving 31 batches of cells transplanted into a total of 215 rats with 6-OHDA lesions [11]. From this study, we found that indeed *LGI1* expression showed a significant enrichment in cell batches with good graft outcome (i.e. high TH + yield in vivo), with a Log2Fold change of 1.15 and an adjusted p-value of 0.037. In contrast, CORIN, showed a strong enrichment in cell batches with poor graft outcome (i.e. low TH + yield in vivo) with a Log2Fold change of -2.31 and an adjusted p-value of 4.92 × 10–8 (data extracted from Additional file 1: Table S2 in [11]). Interestingly, both SERPINF1 and TFF3 also showed significant correlation to poor graft outcome, indicating that these markers should not be used as positive QC markers on d16, whereas CNTN2, CPE, FST and PDGFC did not show significant correlations to good or poor graft outcome in this previous study. Collectively, using the data presented in this study together with the previous correlation analysis, we recommend an ELISA panel assessing the ratio of LGI1 over CORIN as a relevant QC assay to predict correct patterning and in vivo efficacy of cVM cell batches before transplantation.

## Discussion

As several stem cell-derived products for cell replacement therapies have entered clinical trials, there is an increasing need to implement non-invasive GMP compatible assays to provide quality control screening of the in vitro differentiated products during manufacturing. For decades, shotgun proteomics has allowed for unbiased identification and quantification of thousands of proteins, aiding in drug discovery and diagnostics [38–40]. We show here that by applying this approach on the supernatant of cell preparations with clinical relevance to the treatment of Parkinson’s Disease, we could identify novel QC markers which could readily be measured and validated by ELISA.

It is an important feature of our newly identified marker (LGI1), that it can discriminate between rVM and cVM cultures, as these neighbouring regions are normally difficult to discriminate due to their extreme similarity in gene and protein expression patterns [10, 11]. TFF3 was previously identified as a VM-specific marker through an unbiased transcriptomic comparison to dorsal forebrain (dFB)-patterned ESCs [35], and also in our study it showed significantly increased protein and mRNA levels in VM cultures compared to dFB cultures, but was yet indistinguishable between rVM and cVM cultures. Furthermore, TFF3 showed negative correlation to good graft outcome [11], and therefore might not be an optimal marker for monitoring cVM patterning. Our data also showed that the floor plate maker CORIN [41] was markedly elevated in rVM supernatant in comparison to the cVM, and inversely correlated with the expression levels of *EN1*, a *bona fide* indicator of authentic DA VM progenitors for PD therapy [11, 14, 37]. This is of interest given that an ongoing clinical trial in Japan applies flow cytometric purification of CORIN-positive progenitor cells for transplantation to the brains of PD patients [42, 43]. Similarly, CNTN2, has also previously been associated to LMX1A-GFP-sorted VM ESC-derived cells [44], but we show here that this marker is mainly enriched in the non-DA rVM progenitors.

For rapid QC of manufactured cVM DA progenitors, we propose to survey a conjugation of two (or more) reliable protein markers in the supernatant. Our data shows that the ratio between two secreted proteins, one rVM marker and one cVM marker, can readily discriminate rVM from cVM cultures and could be applied to predict the quality of clinical-grade quality-controlled batches of DA cVM progenitors for the STEM-PD trial [45]. Both positive markers of rVM cultures, CNTN2 and CORIN, could be used to clearly discriminate the rVM and cVM culture supernatants by ELISA, and were strongly negatively correlated with *EN1* expression. CNTN2, however, unlike CORIN, did not discriminate at a transcriptional level, and required sample dilution testing, an extra procedure prior to the QC assay. The novel cVM marker FST, positively correlated with *EN1* expression, and could also discriminate rVM from cVM cultures as a FST/CORIN or FST/CNTN2 ratio, even though FST on its own was unable to distinguish the two VM cultures. The surprising disagreement between the FST hits on all MS analyses and the poor discriminative power of FST ELISA results serves as a stark reminder of the need for thorough marker validation by orthogonal assays. Likewise, SERPINF1 could also not be confirmed as a differentially expressed marker by ELISA, even though identified in the MS analysis. The additional novel positive markers for cVM, LGI1 and PDGFC, were found to be highly predictive of cVM cultures, elevated both transcriptionally and in the respective supernatants, and with high correlation to *EN1* expression. The use of these markers in a ratio configuration with either CORIN or CNTN2 yielded the most stringent distinction between the two cell cultures. Altogether, our data points to LGI1/CORIN ratio as the most promising QC assay, as both proteins are particularly elevated in their respective VM region, while being very lowly present or even absent in other non-VM neural progenitor populations and in undifferentiated hESCs. It should be noted that secreted markers cannot stand alone in the QC of cell products, as they do not provide a definite quantitative assay to assess purity or impurity of the cell product in the form of percentages. However, whereas quantitative cell composition assays such as ICC or flow cytometry require harvesting of the cell product, assessment of secreted markers has the advantage that it is an orthogonal assay which can be performed repeatedly as in-process quality control during GMP cell manufacturing without disturbing the cells. Such an assay can therefore serve as a useful go/no-go decision assay during critical steps of cell manufacturing.

Overall, our results showed that the identification of novel cell therapy QC markers through proteomic exploration can aid in the establishment of GMP compliant assays critical for the regulatory assessment of these cell products on their way towards the clinic.

## Conclusions

As hPSC-based cell replacement therapies for PD reach a clinical setting, it is essential to establish stringent multi-factorial QC parameters for the clinically relevant cell products, capable of providing a safeguard against undesired outcomes during manufacturing. Here, we presented a non-invasive, coupled ELISA assay, capable of qualifying GMP-grade DA VM progenitors during differentiation.

### Supplementary Information

**Additional file 1. Fig. S1**. GO term enrichment and peptide detection in MS analyses. **a** GO Term enrichment analysis for the top 10 differentially enriched terms under “Cellular Component” between the Global Secretome DIA and Vesicle DIA samples. **b** Bar graph showing the number of peptides unique for extracellular vesicle markers, for Experiment 3, identified in the Global Secretome DIA and Vesicle DIA samples. **c** Bar graphs showing individual peptide detection rates for 5 selected MS candidate proteins differentially detected between rVM and cVM samples, in the Global Secretome DIA analysis. Note that some peptides where not detected in one condition (absent bars); overall Protein detection rate on top. **d** Bar graphs showing individual peptide detection rates for 3 selected MS candidate proteins differentially detected between rVM and cVM samples, in the Vesicle DIA analysis. Note that some peptides where not detected in one condition (absent bars); overall Protein detection rate on top. **Fig. S2**. Validation of regional batches and temporal assessment of secreted markers. **a** Normalized mRNA expression of the panel for regional markers, assessing the patterning of the samples obtained for qRT-PCR and ELISA analysis of the selected canditates. **b** Quantification of supernatant proteins at day 11 and day 16 of differentiation in cVM and cVM cultures by ELISA. Pairwise comparison within day 11 (rVM and cVM), or between day 11 and day 16 (rVM or cVM) are shown. **Fig. S3**. ELISA measurements of protein ratios against TFF3, CNTN2 and CORIN. **a** Protein quantification ratios between the selected markers and TFF3, in supernatant samples of rVM and cVM cultures at day 16 of differentiation. **b** Protein quantification ratios between the selected markers and CNTN2, in supernatant samples of rVM and cVM cultures at day 16 of differentiation. **c** Protein quantification ratios between the selected markers and CORIN, in supernatant samples of rVM and cVM cultures at day 16 of differentiation. **Table S1:** List of human primers, by gene, full name, and forward and reverse sequence. **Table S2:** List of ELISA kits. The table shows manufacturer, kit name and catalog numbers for ELISA kits used in this study. **Table S3:** Extracellular vesicle markers in rVM and cVM samples in Experiment 3a+b. List of non-tissue specific extracellular vesicle associated proteins identified by MS-DIA in Experiment 3, in the Global Secretome samples and ultracentrifuged Vesicle-enriched samples, comparing cVM and rVM cultures. No markers showed a fold change larger than ±2, and changes were therefore regarded as non-significant ND: not detected, EV: extracellular vesicles. P-value = sca.P.value, q-value = sca.adj.pval. 

**Additional file 2. Table S4:** Values from all MS differential expression analysis for all experiments.

## Data Availability

The mass spectrometry proteomics data has been deposited to the ProteomeXchange Consortium (www.proteomexchange.org/) via the PRIDE partner repository [46], with the dataset identifier PXD039510.

## Abbreviations

AGC: Automatic gain control
BSA: Bovine serum albumin
cVM: Caudal ventral midbrain
DA: Dopaminergic
DDA: Data-dependent acquisition
dFB: Dorsal forebrain
DIA: Data-independent acquisition
dHB: Dorsal hindbrain
dMB: Dorsal midbrain
DTT: Dithiothreitol
ELISA: Enzyme-linked immunosorbent assay
GPF: Gas-phase fractionation
GMP: Good manufacturing practices
hPSC: Human pluripotent stem cell
IAA: Iodoacetamide
IT: Ion injection time
LC: Liquid chromatography
LFQ: Label-free quantification
MS: Mass spectrometry
MS1: Precursor ion spectra
MS2: Fragment ion spectra
NCE: Normalized collision energy
PD: Parkinson’s disease
QC: Quality control
qRT-PCR: Quantitative reverse transcription polymerase chain reaction
rVM: Rostral ventral midbrain
SHH: Sonic hedgehog
vFB: Ventral forebrain
vHB: Ventral hindbrain
VM: Ventral midbrain
