# Supplementary material for: Identifying secreted biomarkers of dopaminergic ventral midbrain progenitor cells
Source: Stem Cell Res Ther. 2023 Dec 10;14:354. doi: 10.1186/s13287-023-03580-5 (PMC10712201; doi:10.1186/s13287-023-03580-5)
Supplement: Supplementary file 1 — Additional file 1. Fig. S1. GO term enrichment and peptide detection in MS analyses. a GO Term enrichment analysis for the top 10 differentially enriched terms under “Cellular Component” between the Global Secretome DIA and Vesicle DIA samples. b Bar graph showing the number of peptides unique for extracellular vesicle markers, for Experiment 3, identified in the Global Secretome DIA and Vesicle DIA samples. c Bar graphs showing individual peptide detection rates for 5 selected MS candidate proteins differentially detected between rVM and cVM samples, in the Global Secretome DIA analysis. Note that some peptides where not detected in one condition (absent bars); overall Protein detection rate on top. d Bar graphs showing individual peptide detection rates for 3 selected MS candidate proteins differentially detected between rVM and cVM samples, in the Vesicle DIA analysis. Note that some peptides where not detected in one condition (absent bars); overall Protein detection rate on top. Fig. S2. Validation of regional batches and temporal assessment of secreted markers. a Normalized mRNA expression of the panel for regional markers, assessing the patterning of the samples obtained for qRT-PCR and ELISA analysis of the selected canditates. b Quantification of supernatant proteins at day 11 and day 16 of differentiation in cVM and cVM cultures by ELISA. Pairwise comparison within day 11 (rVM and cVM), or between day 11 and day 16 (rVM or cVM) are shown. Fig. S3. ELISA measurements of protein ratios against TFF3, CNTN2 and CORIN. a Protein quantification ratios between the selected markers and TFF3, in supernatant samples of rVM and cVM cultures at day 16 of differentiation. b Protein quantification ratios between the selected markers and CNTN2, in supernatant samples of rVM and cVM cultures at day 16 of differentiation. c Protein quantification ratios between the selected markers and CORIN, in supernatant samples of rVM and cVM cultures at day 16 of differentiation. Tabl [file 13287_2023_3580_MOESM1_ESM.pdf]

Supplementary Figure 1

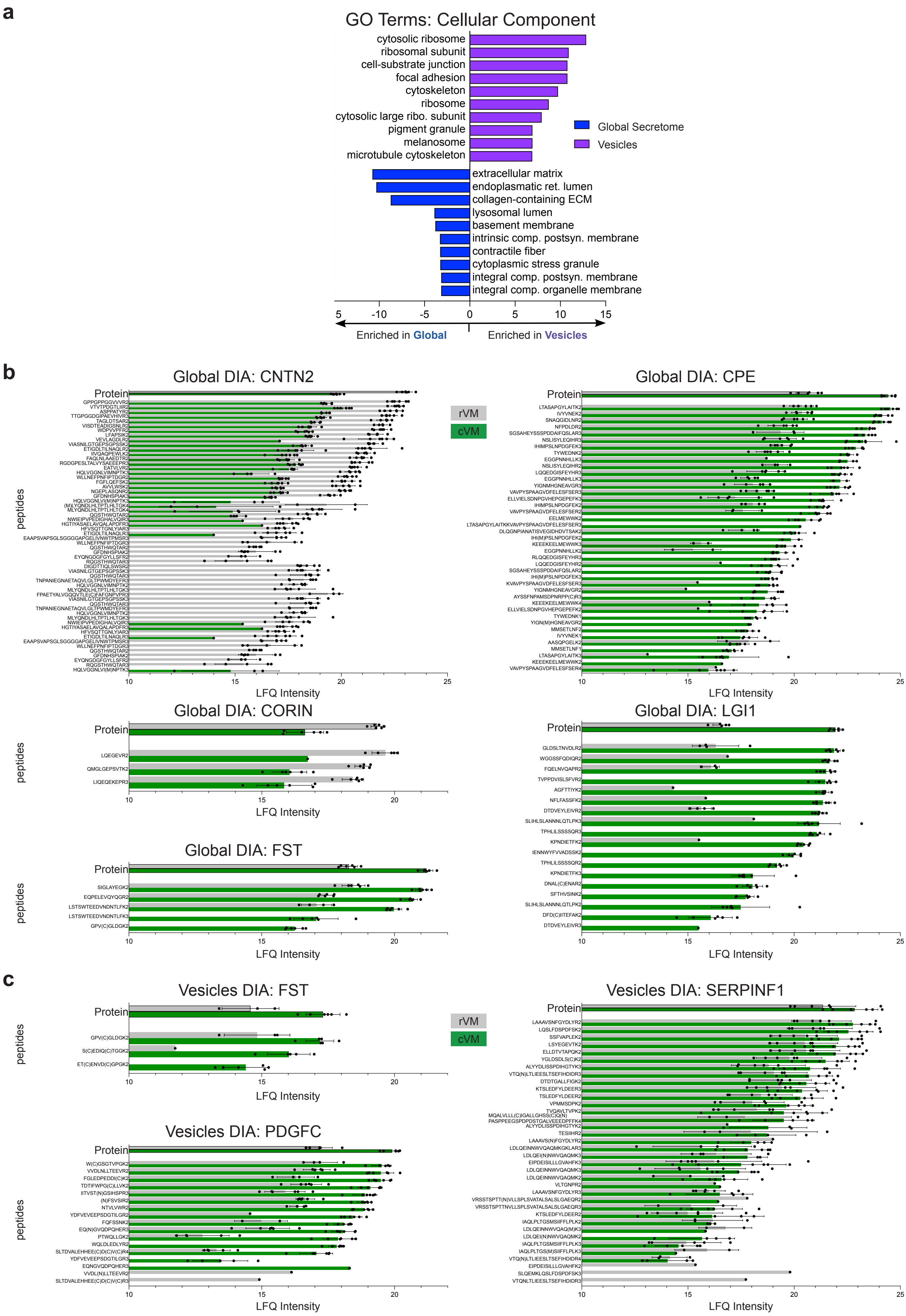

Figure S1. GO term enrichment and peptide detection in MS analyses
a) GO Term enrichment analysis for the top 10 differentially enriched terms under “Cellular Component” between the Global Secretome DIA and Vesicle DIA samples.
b) Bar graph showing the number of peptides unique for extracellular vesicle markers, for Experiment 3, identified in the Global Secretome DIA and Vesicle DIA samples.
c) Bar graphs showing individual peptide detection rates for 5 selected MS candidate proteins differentially detected between rVM and cVM samples, in the Global Secretome DIA analysis. Note that some peptides where not detected in one condition (absent bars); overall Protein detection rate on top.
d) Bar graphs showing individual peptide detection rates for 3 selected MS candidate proteins differentially detected between rVM and cVM samples, in the Vesicle DIA analysis. Note that some peptides where not detected in one condition (absent bars); overall Protein detection rate on top.

Supplementary Figure 2

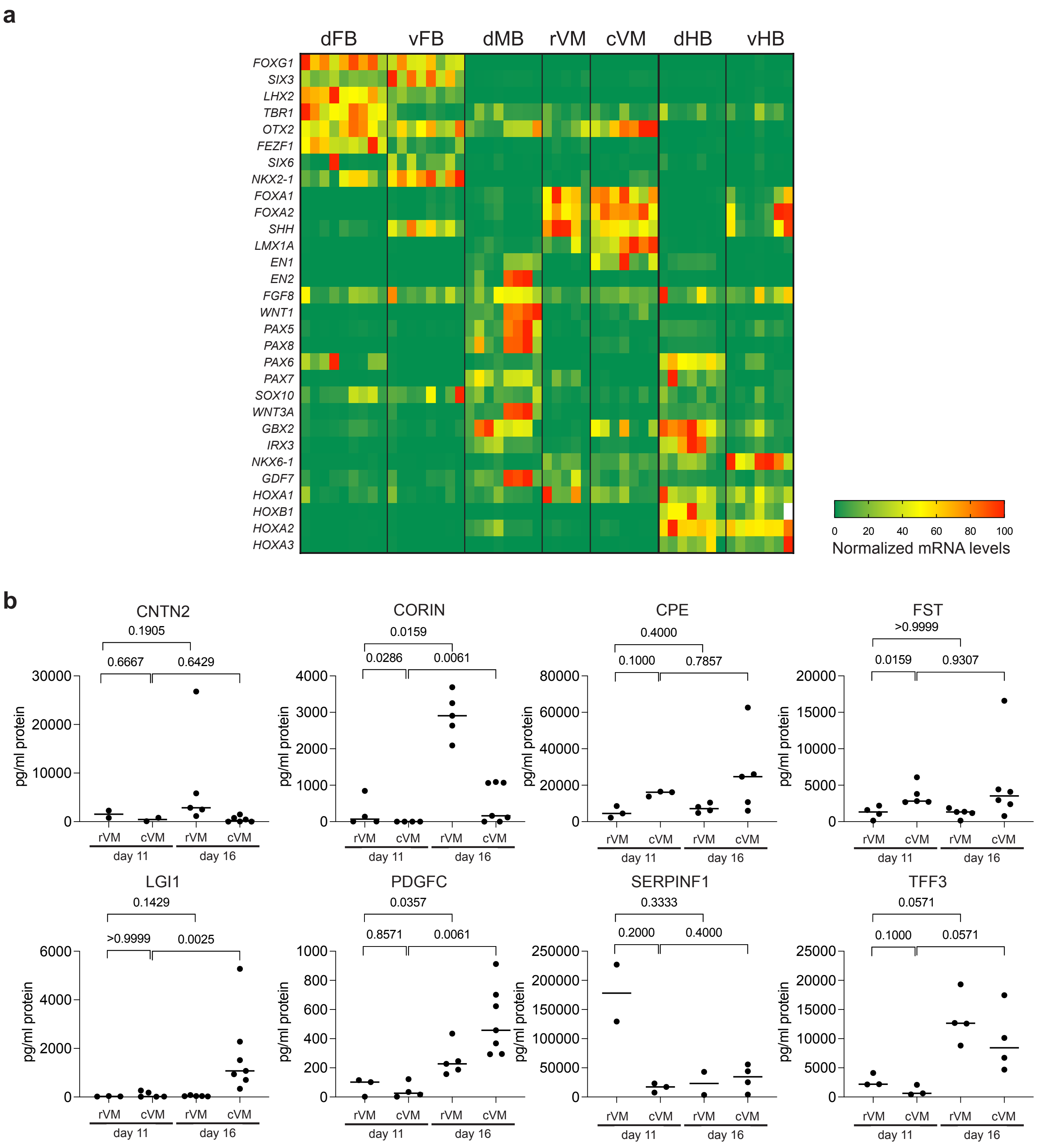

Figure S2. Validation of regional batches and temporal assessment of secreted markers
a) Normalized mRNA expression of the panel for regional markers, assessing the patterning of the samples obtained for qRT-PCR and ELISA analysis of the selected canditates.
b) Quantification of supernatant proteins at day 11 and day 16 of differentiation in cVM and cVM cultures by ELISA. Pairwise comparison within day 11 (rVM and cVM), or between day 11 and day 16 (rVM or cVM) are shown.

Supplementary Figure 3

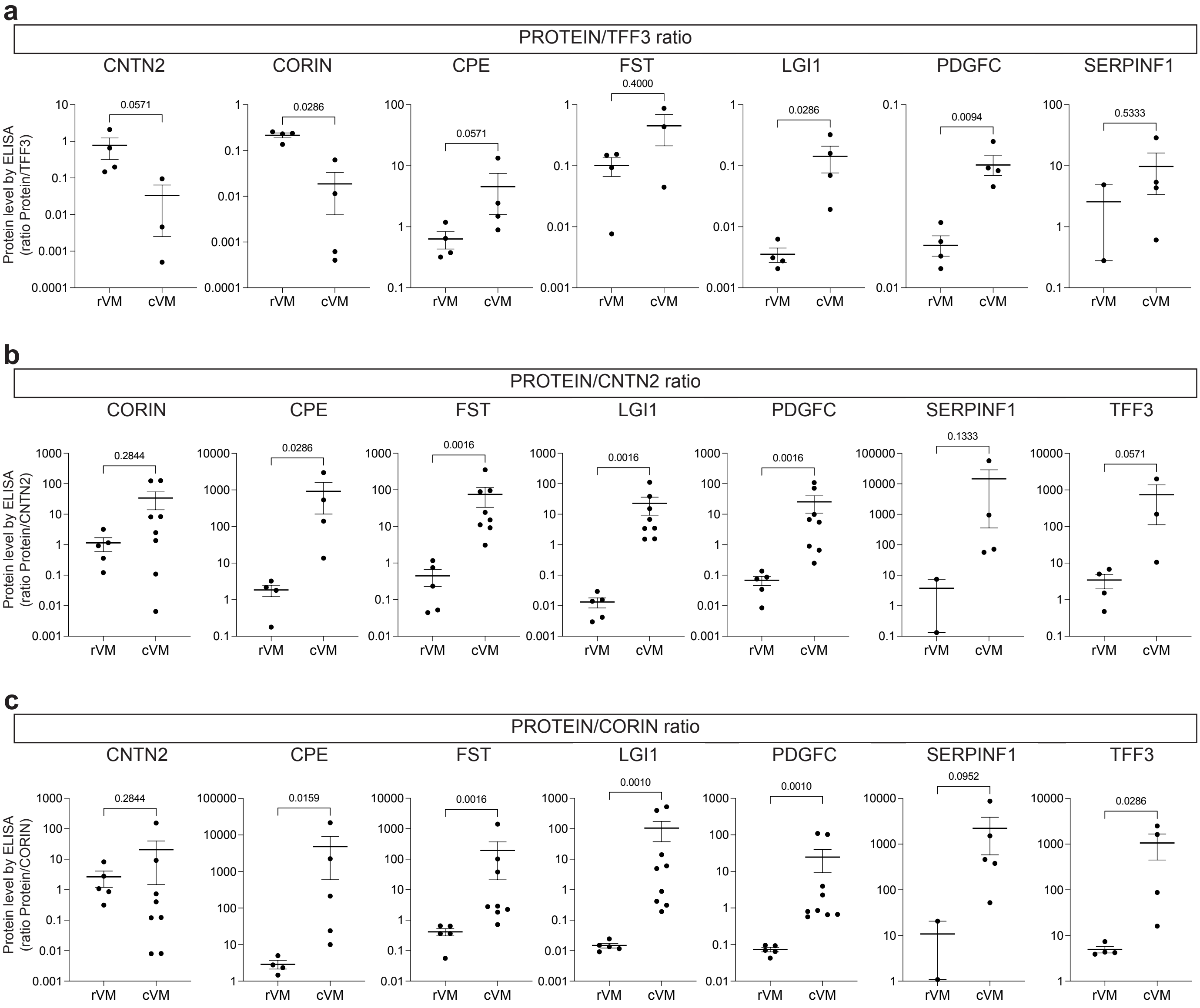

Figure S3. ELISA measurements of protein ratios against TFF3, CNTN2 and CORIN

a) Protein quantification ratios between the selected markers and TFF3, in supernatant samples of rVM and cVM cultures at day 16 of differentiation.

b) Protein quantification ratios between the selected markers and CNTN2, in supernatant samples of rVM and cVM cultures at day 16 of differentiation.

c) Protein quantification ratios between the selected markers and CORIN, in supernatant samples of rVM and cVM cultures at day 16 of differentiation.

Primer	Gene (full name)	Forward Primer	Reverse Primer
ACTB	actin beta	CCTTGCACATGCCGGAG	GCACAGAGCCTCGCCTT
CNTN2	contactin 2	GTCACGGGAGTACCAGAACG	TGTAGACAAAGTACTGGGCATCG
CORIN	corin, serine peptidase	CATATCTCCATCGCCTCAGTTG	GGCAGGAGTCCATGACTGT
CPE	carboxypeptidase E	CTCTGAAGACCTACTGGGAGGA	GCATTCGCAATTGGGTTACCTT
EN1	engrailed homeobox 1	CGTGGCTTACTCCCCATTTA	TCTCGCTGTCTCTCCCTCTC
EN2	engrailed homeobox 2	CCTCCTGCTCCTCCTTTCTT	GACGCAGACGATGTATGCAC
FEZF1	FEZ family zinc finger 1	GGTACATTCCACATTCGTGAGC	TCACGTGCAATAATCAAAACCA
FGF8	fibroblast growth factor 8	ACAGCGCTGCAGAATGCCAAGT	GAAGTGGACCTCACGCTGGTGC
FOXA1	forkhead box A1	GGGCAGGGTGGCTCCAGGAT	TGCTGACCGGGACGGAGGAG
FOXA2	forkhead box A2	CCGTTCTCCATCAACAACCT	GGGGTAGTGCATCACCTGTT
FOXG1	forkhead box G1	TGGCCCATGTCGCCCTTCCT	GCCGACGTGGTGCCGTTGTA
FST	follistatin	GATGGGAAAACCTACCGCAATG	CATCTGCCTTGGTACTGGACTT
GAPDH	glyceraldehyde-3-phosphate dehydrogenase	TTGAGGTCAATGAAGGGGTC	GAAGGTGAAGGTCGGAGTCA
GBX2	gastrulation brain homeobox 2	GTTCCCGCCGTCGCTGATGAT	GCCGGTGTAGACGAAATGGCCG
GDF7	growth differentiation factor 7	GACGCTGCTCAACTCCATGGCA	TTGGCGGCGTCGATGTAGAGGA
HOXA1	homeobox A1	GTACGGCTACCTGGGTCAAC	ACTTGGGTCTCGTTGAGCTG
HOXA2	homeobox A2	CGTCGCTCGCTGAGTGCCTG	TGTCGAGTGTGAAAGCGTCGAGC
HOXA3	homeobox A3	GGCCAATCTGCTGAACCTCA	GAGTTCAGATAGCCACCGGC
HOXB1	homeobox B1	GGCCTTCTCAGTACTACCCTCT	CCGTAGCTCGAGGGATGAAAAT
IRX3	iroquois homeobox 3	GGCTTGCGCCCCGTAGAAATGT	AGGAGCCAGGTCAGGTCCGAAC
LGI1	leucine rich glioma inactivated 1	CAACAATCTCCAGACACTCCCA	CCCCTCAGGTCCACATTTGTTA
LHX2	LIM homeobox 2	GGGCGACCACTTCGGCATGAA	CGTCGGCATGGTTGAAGTGTGC
LMX1A	LIM homeobox transcription factor1a	CGCATCGTTTCTTCTCCTCT	CAGACAGACTTGGGGCTCAC
NKX2-1	NK2 homeobox 1	AGGGCGGGGCACAGATTGGA	GCTGGCAGAGTGTGCCCAGA
NKX6-1	NK6 homeobox 1	GGATCCCAACTCGGACGACGAGA	AGGATGAGCTCTCCGGCTCGG
OTX2	orthodenticle homeobox 2	ACAAGTGGCCAATTCACTCC	GAGGTGGACAAGGGATCTGA
PAX5	paired box 5	CCCCATTGTGACAGGCCGTGAC	TCAGCGTCGGTGCTGAGTAGCT
PAX6	paired box 6	TGGTATTCTCTCCCCCTCCT	TAAGGATGTTGAACGGGCAG
PAX7	paired box7	CTTCAGTGGGAGGTCAGGTT	CAAACACAGCATCGACGG
PAX8	paired box 8	ATAGCTGCCGACTAAGCATTGA	ATCCGTGCGAAGGTGCTTT
PDGFC	platelet derived growth factor C	ACAAGGAACAGAACGGAGTACA	GTATGAGGAAACCTTGGGCTGT
SERPINF1	serpin family F member 1	TCGGACCCTAAGGCTGTTTTAC	CTTTCAGGGGCAGGAAGAAGAT
SHH	sonic hedgehog	CCAATTACAACCCCGACATC	AGTTTCACTCCTGGCCACTG
SIX3	SIX homeobox 3	ACCGGCCTCACTCCCACACA	CGCTCGGTCCAATGGCCTGG
SIX6	SIX homeobox 6	CTCAACAAGAATGAGTCGGTGC	ACTCCTTGGTGAACTTGTGGTT
SOX10	SRY-box 10	CTTTCTTGTGCTGCATACGG	AGCTCAGCAAGACGCTGG
TBR1	T-box, brain 1	TCGTCCCCGCTCAAGAGCGA	CCTTGGCGCAGTTCTTCTCGCA
TFF3	trefoil factor 3	TCTGGAGCCTGATGTCTTAACG	GACGCAGCAGAAATAAAGCACA
WNT1	Wnt family member 1	GAGCCACGAGTTTGGATGTT	TGCAGGGAGAAAGGAGAGAA
WNT3A	Wnt family member 3A	GCGATGGCCCCACTCGGATACT	TAGCTGCCCAGAGCCTGCTTCA

Table S1: List of human primers, by gene, full name, and forward and reverse sequence

Protein	Kit Manufacturer	Kit name	Kit target	Catalog #
**CNTN2**	R & D Systems	DuoSet	human Contactin-2/TAG1	DY1714-05
**CORIN**	R & D Systems	Quantikine	human Corin	DCRN00
**CPE**	Nordic Biosite	n.a.	human CPE	KBB-B314QW-96
**FST**	R & D Systems	Quantikine	human Follistatin	DFN00
**LGI1**	Cusabio	n.a.	human LGI1	CSB-EL012898HU
**PDGFC**	R & D Systems	Quantikine	human PDGF-CC	DCC00
		DuoSet		DY1687-05
**SERPINF1**	R & D Systems	DuoSet	human SerpinF1/PEDF	DY1177-05
**TFF3**	R & D Systems	Quantikine	human TFF3	DTFF30

Table S2: List of ELISA kits
The table shows manufacturer, kit name and catalog numbers for ELISA kits used in this study

	Experiment 3a: Global DIA			Experiment 3b: Vesicles DIA		
EV markers	fold change (cVM/rVM)	p-value	q-value	fold change (cVM/rVM)	p-value	q-value
PDCD6IP (ALIX)	0,10	0,837	0,887	-1,28	1,27E-06	2,46E-05
TSG101	ND	ND	ND	-1,52	2,40E-06	3,69E-05
CD63	ND	ND	ND	-0,59	0,013	0,033
CD81	ND	ND	ND	-0,62	0,015	0,038
CD47	ND	ND	ND	-1,52	2,04E-09	4,80E-07
VPS4B	0,40	0,235	0,325	-1,00	9,39E-05	6,05E-04

Table S3: Extracellular vesicle markers in rVM and cVM samples in Experiment 3a+b
List of non-tissue specific extracellular vesicle associated proteins identified by MS-DIA in Experiment 3, in the Global Secretome samples and ultracentrifuged Vesicle-enriched samples, comparing cVM and rVM cultures. No markers showed a fold change larger than ±2, and changes were therefore regarded as non-significant ND: not detected, EV: extracellular vesicles. P-value = sca.P.value, q-value = sca.adj.pval
